# Anticancer Activity of Benzomalvin Derivatives Isolated from *Penicillium spathulatum* SF7354, a Symbiotic Fungus from *Azorella monantha*

**DOI:** 10.4014/jmb.2505.05003

**Published:** 2025-09-16

**Authors:** Min Seo Jeon, Ju-Mi Hong, Jaewon Kim, Jina Kim, Sojin Kim, Jae Hak Sohn, Se Jong Han, Joung Han Yim, Il-Chan Kim

**Affiliations:** 1 Division of Polar Life Sciences, Korea Polar Research Institute, Incheon 21990, Republic of Korea; 2 Department of Biomedical Laboratory Science, Daegu Health College, Daegu 41453, Republic of Korea; 3 Division of Bioindustry, College of Medical and Life Sciences, Silla University, Busan, 46958, Republic of Korea

**Keywords:** Patagonia, *Penicillium spathulatum*, anticancer, apoptosis, benzomalvin

## Abstract

*Penicillium spathulatum* SF7354 was isolated from the extremophilic plant *Azorella monantha* collected in Chilean Patagonia and investigated for its anticancer potential. Crude extracts of SF7354 exhibited significant cytotoxic activity against multiple human cancer cell lines, with the most pronounced effect observed in HCT116 cells. Flow cytometry analysis revealed that treatment with the extract induced time-dependent apoptosis and sub-G1 accumulation, indicating activation of programmed cell death. Cell cycle analysis further showed early G0/G1 arrest, followed by a progressive increase in apoptotic populations. Western blot analysis demonstrated notable alterations in PARP and p53 protein levels, suggesting a p53-dependent mechanism of apoptosis. HPLC-based purification of the extract led to the isolation of five benzomalvin derivatives (A–E), all of which exhibited dose- and time-dependent cytotoxicity. These findings suggest that SF7354-derived benzomalvins act through apoptosis-associated mechanisms and represent promising candidates for the development of novel anticancer agents.

## Introduction

Patagonia, located at the southern end of South America, is a geographically and climatically diverse region known for its extreme environmental conditions, including high UV radiation, strong winds, low temperatures, and nutrient-poor soils [1]. These harsh conditions have driven the evolution of unique biological communities, particularly among microorganisms and plants associated with extremophiles [2]. Many native Patagonian plants have developed symbiotic relationships with endophytic fungi and bacteria, which may contribute to their host’s adaptability by producing secondary metabolites with protective or regulatory functions [3]. This makes the Patagonian ecosystem a valuable reservoir for the discovery of new secondary metabolites, particularly in the context of increasing antimicrobial resistance and the urgent need for novel drug leads.

Among these resilient plant species, *Azorella monantha*—a cushion plant endemic to Patagonia—plays an important ecological role and has been recognized as a potential reservoir for symbiotic microorganisms [4]. Recent studies have suggested that the microbial communities associated with *Azorella* species harbor promising biosynthetic potential, particularly fungi capable of producing pharmacologically active compounds [5]. Endophytes isolated from *Azorella* have shown antimicrobial, antioxidant, and anti-inflammatory activities, raising the possibility that such microbes may also possess anticancer properties.

*Penicillium* spp. are one of the most widely studied fungal genera in the context of secondary metabolite production, including antibiotics, mycotoxins, and anticancer agents [6]. Fungi of this genus have been reported to synthesize a broad spectrum of bioactive molecules such as polyketides and alkaloids, many of which exhibit cytotoxicity against human cancer cells [7]. In this study, we report the isolation of a *Penicillium* strain from *A. monantha* growing in the pristine regions of Patagonia and investigate its potential anticancer activity against HCT116 cells. This work aims to explore the underexploited microbial diversity of Patagonia as a source of novel chemotherapeutic agents.

## Materials and Methods

### Microbial Isolation from *Azorella monantha*

Samples of *A. monantha* were collected from the Magallanes Region of Chilean Patagonia (53°39'00''S, 70°57'41''W) and transported to the laboratory under sterile conditions. Plant segments were homogenized using a sterilized mortar and pestle under aseptic conditions. The resulting homogenates were serially diluted with sterile distilled water and spread onto potato dextrose agar (PDA) plates [8]. The plates were incubated at 10°C for 30 days to allow microbial growth. The isolates were purified and identified based on morphological characteristics and molecular analyses using ITS sequencing.

### Isolation of Fungal Secondary Metabolites

The fungal isolates were incubated in potato dextrose broth (PDB) at 15°C for 21 days with agitation at 120 rpm. The culture broth was subjected to liquid–liquid partitioning and extracted three times with an equal volume of ethyl acetate. The combined organic layers were concentrated under reduced pressure using a rotary evaporator.

The crude extract was initially fractionated using a medium-pressure liquid chromatography (MPLC) system (EPCLC-AI-580S, Yamazen, Japan). Gradient elution was performed on a silica gel column using a hexane (A)–ethyl acetate (B) solvent system. The following gradient was applied: 11% solvent B for the initial 7 min, followed by a linear increase to 40% B over the next 20 min. The composition was then held at 40% B for 10 min, ramped to 100% B over 13 min, and maintained at 100% B for the final 20 min. Extracts were further fractionated using an ODS column (12.3 × 2.3 mm, 120 Å, Yamazen) and MPLC system.

Extracts were further fractionated using an ODS column (12.3 × 2.3 mm, 120 Å, Yamagen) and MPLC system. The first-round fractionated sample was further purified under the following conditions. A mobile phase consisted of water (A) and methanol (B). The gradient elution was programmed as follows: 5% B for 3 min, a linear increase to 50% B over 10 min, and finally 100% B for 5 min. The target fraction of extracts was collected and further purified under the following conditions. The gradient program employed water (A) and methanol (B) as the mobile phase, with the following steps: 29% B for 3 min, linear increase to 50% B over 10 min, held at 50% B for 10 min, followed by a linear increase to 82% B over 15 min, held at 82% B for 10 min, then increased to 100% B over 15 min and held for an additional 10 min.

Subsequently, target fraction was purified by semi-preparative HPLC using 0.1% formic acid in water (A) and 0.1% formic acid in acetonitrile (B), performed on a Vanquish system (Thermo Fisher Scientific, USA). The gradient elution profile was as follows: 5% B (2 min), 5–40% B (2 min), 40–80% B (22 min), 80–100% B (0.5 min), 100% B (5 min), 100–5% B (0.5 min), 5% B (5 min), with a total run time of 35 min. Each single compound was identified based on MS/MS data.

### Cell Culture

A549, HeLa, Hs578T, Huh7, A375, and HCT-116 cells were maintained in Dulbecco’s Modified Eagle Medium (DMEM) supplemented with 10% heat-inactivated fetal bovine serum (FBS) and 1% penicillin/streptomycin. Cells were grown in a culture dish at 37°C in a humidified atmosphere containing 5% CO_2_ and 95% air.

### Cell Viability Analysis

Cancer cells were seeded in 96-well plates at a density of 1×10^5^ cells/ml and incubated with various concentrations of crude extracts for 24 h. Cell viability was assessed based on the mitochondria-dependent reduction of 3-(4,5-dimethyl-2-thiazolyl)-2,5-diphenyl-2H-tetrazolium bromide (MTT) to formazan. Briefly, 5 μl of MTT solution (5 mg/ml) was added to each well, followed by incubation for 4 h at 37°C. After removing the medium, dimethyl sulfoxide (DMSO) was added to dissolve the formazan crystals. The absorbance of the resulting solution was measured at 570 nm using a microplate reader (Thermo Fisher Scientific Inc.). Doxorubicin (DOC) at a concentration of 1 mM was used as positive control. Cell viability was calculated as a percentage relative to the untreated control, which was set as 100% [9].

To evaluate the effect of the fractionated extract on HCT116 cells, the cells were treated with various concentrations of the extract. Cell viability was assessed at 24, 48, and 72 h post-treatment using the MTT assay. Morphological changes were examined using an inverted phase contrast microscope (EVOS, USA)

### Flow Cytometric Analysis

Apoptosis and/or necrosis in HCT116 cells was assessed using Annexin V/fluorescein isothiocyanate (FITC) and propidium iodide (PI) double staining. Briefly, 1 × 10^5^ cells/ml were seeded into 6-well plates and treated with 20 μg/ml of the fractionated extract. After incubation, the cells were washed, harvested, and stained with Annexin V/FITC and PI (BD Biosciences, USA) following the manufacturer's instructions. Untreated cells stained with either Annexin V/FITC or PI were used as controls. The samples were analyzed using a flow cytometer (Beckman Coulter Inc., USA). Cell cycle analysis was performed as described previously [10].

### qRT-PCR Confirmation of Differentially Expressed Genes

To validate the *in silico* analysis of differentially expressed genes, quantitative real-time PCR (qRT-PCR) was performed. Total RNA was extracted from HCT116 cells using TRIzol reagent (Invitrogen, USA) according to the manufacturer’s protocol. The extracted RNA was then directly subjected to qRT-PCR using the Luna Universal One-Step SYBR Green RT-qPCR Kit (New England Biolabs Inc., USA). qRT-PCR was performed using a QuantStudio 1 Real-Time PCR System (Applied Biosystems, USA). In each run, transcript levels in treated cells were directly compared to those in the control group. β-actin was used as the reference gene, and each target gene was analyzed in at least three independent experiments. Data from qRT-PCR were analyzed using QuantStudio Design & Analysis Software version 1.5.1 (Applied Biosystems).

### Western Blot Analysis

Cells were lysed with RIPA buffer and constantly agitated for 30 min. The cell lysate was centrifuged in a microcentrifuge at 4°C, and the supernatant was collected in a fresh tube kept on ice. Equal amount of total protein (20 μg) was used for the western blot detection of each target gene. The primary anti-bodies used detected PARP, p53, and ACTB. After probing with secondary antibody conjugated to horseradish peroxidase, the protein signals were detected using film and chemiluminescence. Protein levels of PARP and p53 were examined at 48 and 72 h post-treatment.

### Identification of Fungal Secondary Metabolites

Single compounds were isolated using a semi-preparative high-performance liquid chromatography (HPLC) system (Vanquish, Thermo Fisher Scientific Inc.) with a mobile phase consisting of 0.1% formic acid in water (A) and 0.1% formic acid in acetonitrile (B). An Inspire C18 (250 × 10 mm i.d., 10 μ, USA) was used. Gradient elution was performed as follows: the mobile phase was maintained at 5% solvent B for 2 min, increased linearly to 40% B over 2 min, and then to 80% B over the following 22 min. It was further ramped to 100% B over 0.5 min and held at that composition for 5 min. The gradient was then decreased to 5% B over 0.5 min, followed by re-equilibration at 5% B for an additional 5 min.

Isolated compounds were evaporated in a SpeedVac concentrator at a high-speed drying mode and redissolved in methanol for ultra-performance liquid chromatography–tandem mass spectrometry (UPLC–MS/MS) analysis. The UPLC–MS/MS analysis system consisted of a Triple TOF 4600 system (AB Sciex, USA) equipped with an electrospray ionization (ESI) source in positive ion mode. An Endeavorsil UPLC C18 column (100 × 2.1 mm i.d., 1.8 μ, Dikma, Canada). Mobile phases were 0.1% formic acid in water(A) and 0.1% formic acid in acetonitrile (B). The following gradient program was applied: 5–100% solvent B over 10 minutes, held at 100% B for 2.5 min, decreased to 5% B over 0.5 min, and maintained at 5% B for 2 min. The column oven temperature was maintained at 40°C. The injection volume was 5 μl, and the flow rate was set at 0.3 ml/min. Mass spectra were recorded over an m/z range of 100–2,000.

To evaluate the effect of the single compounds on HCT116 cells, the cells were treated with various concentrations. Cell viability was assessed at 24, 48, and 72 h post-treatment using MTT assay.

### Statistical Analysis

Statistical Analysis System software (SAS Institute Inc., USA) was used. One-way analysis of variance (ANOVA) and Duncan’s multiple range tests were performed to determine statistical significance (*p* < 0.01).

## Results

### Effects of Fungal Extracts on Cancer Cell Viability

Strain SF7354 was isolated from *A. monantha* specimens collected in Patagonia. Analysis of the ITS region revealed that strain SF7354 shares 99.49% similarity with *Penicillium spathulatum* (GenBank accession number KC427190.1). The GenBank accession number for the ITS region of strain SF7354 is PV82620.

**Fig. 1:**
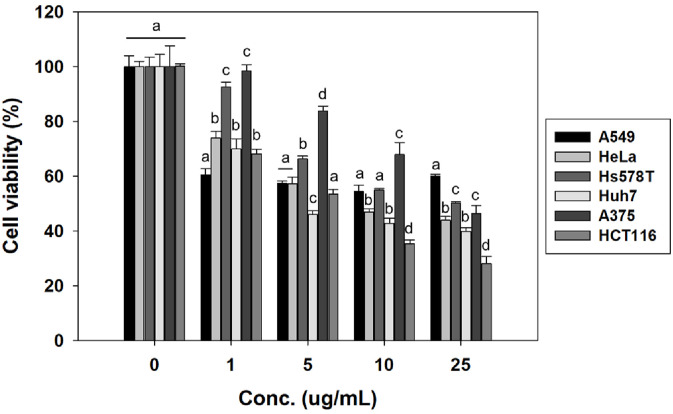
Cytotoxic effects of crude extracts from *Penicillium spathulatum* SF7354 on multiple human cancer cell lines. Cells were treated with various concentrations (1, 5, 10, and 25 μg/ml) of crude extract for 24 h. Cell viability was measured by MTT assay and expressed as a percentage of the untreated control. Data represent mean ± standard error (*n* = 3).

The cytotoxic effects of crude extracts derived from *P. spathulatum* SF7354 were evaluated across six human cancer cell lines (A549, HeLa, Hs578T, Huh7, A375, and HCT116) at concentrations of 1, 5, 10, and 25 μg/ml. A dose-dependent reduction in cell viability was observed in all cell lines, with increasing extract concentrations leading to greater cytotoxicity. Among the tested cell lines, HCT116 cells exhibited the most significant decrease in viability, with only 28.03% survival at the highest concentration (25 μg/ml), suggesting a higher sensitivity to the fungal extract compared to the other cell lines. Based on these results, HCT116 was selected for further investigation into the anticancer properties of strain SF7354 (Fig. 1).

**Fig. 2:**
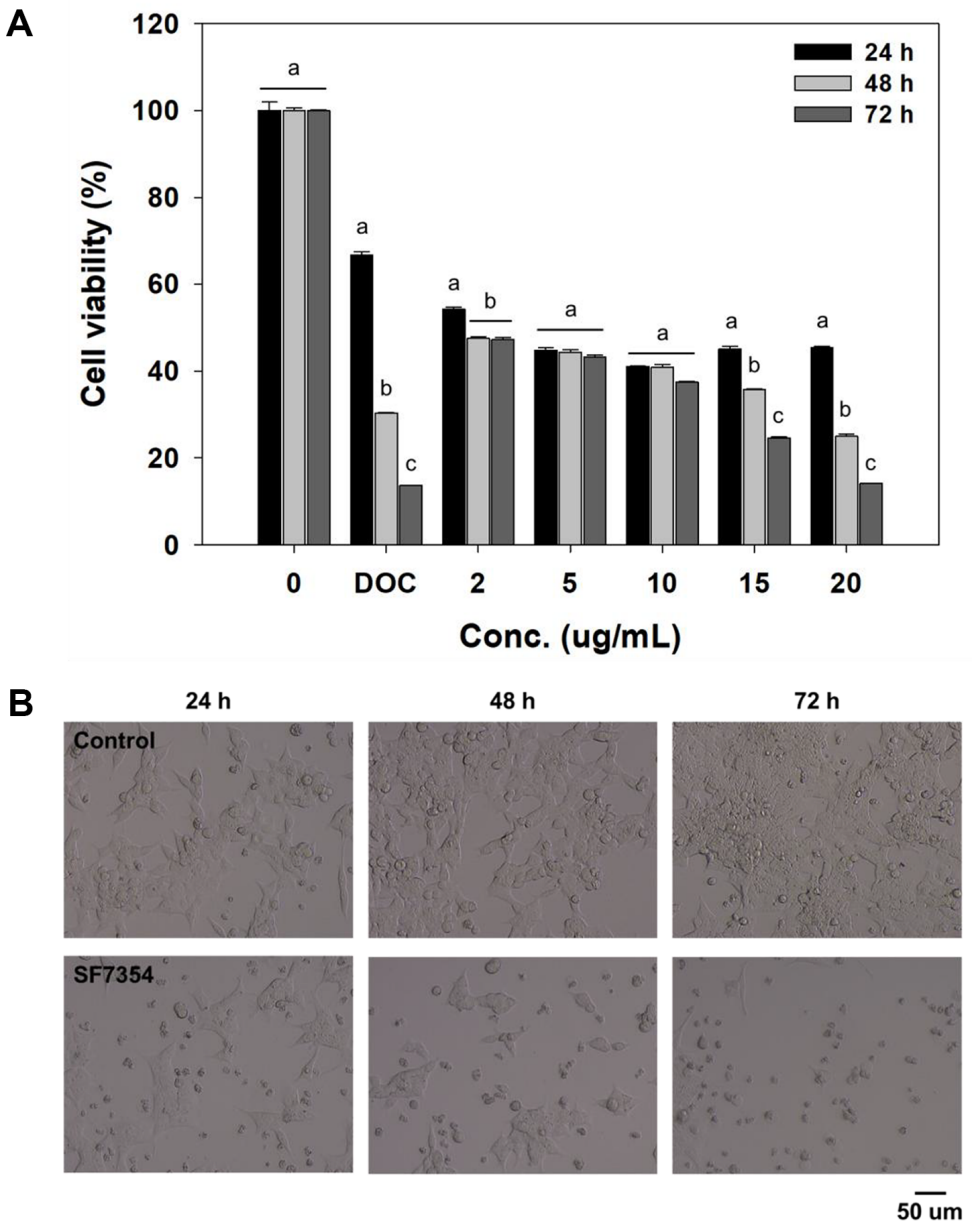
Time- and dose-dependent cytotoxicity of fractionated extracts from *P. spathulatum* SF7354 on HCT116 cells. (**A**) Cell viability was measured by MTT assay after 24, 48, 72 h. Different letters (a-c) indicate significant difference among groups (*p* < 0.01). (**B**) Phase-contrast microscopy revealed normal morphology in control cells, whereas treated cells displayed shrinkage, detachment, and reduced cell density in a dose-dependent manner.

To evaluate the cytotoxic activity of the fractionated extract of strain SF7354, HCT116 cells were treated with various concentrations for 24, 48, and 72 h. As shown in the MTT assay results (Fig. 2), cell viability decreased in a dose- and time-dependent manner. At the highest concentration of 20 μg/ml, viability was significantly reduced to 14.13% after 72 h of treatment, suggesting the potent cytotoxic effect of the extract.

The microscopic examination further confirmed the cytotoxic effects observed in the MTT assay. Compared to the control group, treated cells exhibited morphological changes characteristic of cell death, including cell shrinkage, detachment from the culture surface, and loss of typical cellular structure (Fig. 2B). These findings indicate that the SF7354-derived extract exerts strong antiproliferative activity on HCT116 cells, consistent with the induction of cell death pathways.

### Apoptotic and Cell Cycle Responses of HCT116 Cells to Fungal Extract

**Fig. 3:**
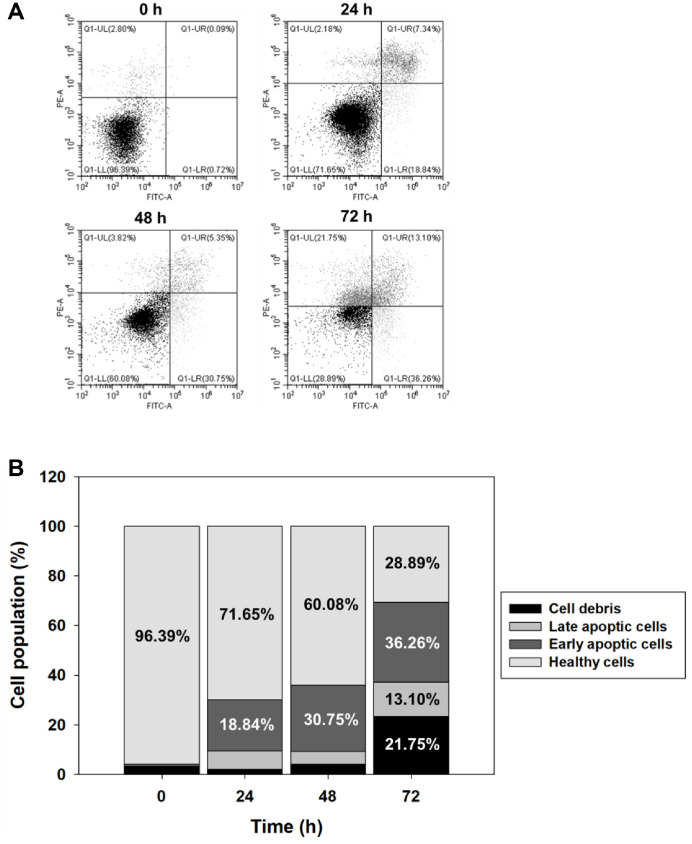
Flow cytometric analysis of apoptosis induction in HCT116 cells by *P. spathulatum* SF7354 extract. (**A**) Apoptosis detection using FITC-labeled Annexin V and PI staining. Apoptotic cells were identified based on increased Annexin V fluorescence. (**B**) Quantification of cell populations. The percentage of apoptotic cells increased in a timedependent manner following treatment, as determined by FACS analysis.

To evaluate the apoptosis-inducing potential of the fungal extract, Annexin V-FITC/PI double staining was performed on HCT116 cells treated with 20 μg/ml of the extract for 24, 48, and 72 h. At 0 h (untreated), the vast majority of cells remained viable (Q1-LL, 96.39%), with negligible populations of early (Q1-LR, 0.72%) or late apoptotic cells (Q1-UR, 0.09%). Upon treatment, a time-dependent increase in apoptotic cell populations was observed. At 24 h, the proportions of early and late apoptotic cells increased to 18.84% and 7.34%, respectively. This trend was more pronounced at 48 h, with early apoptotic cells reaching 30.75% and late apoptotic cells to 5.35%. After 72 h of treatment, early and late apoptotic populations further rose to 36.26% and 13.10%, respectively, while viable cells decreased to 28.89% (Fig. 3). These results indicate that the SF7354-derived extract induces significant apoptosis in HCT116 cells in a time-dependent manner.

**Fig. 4:**
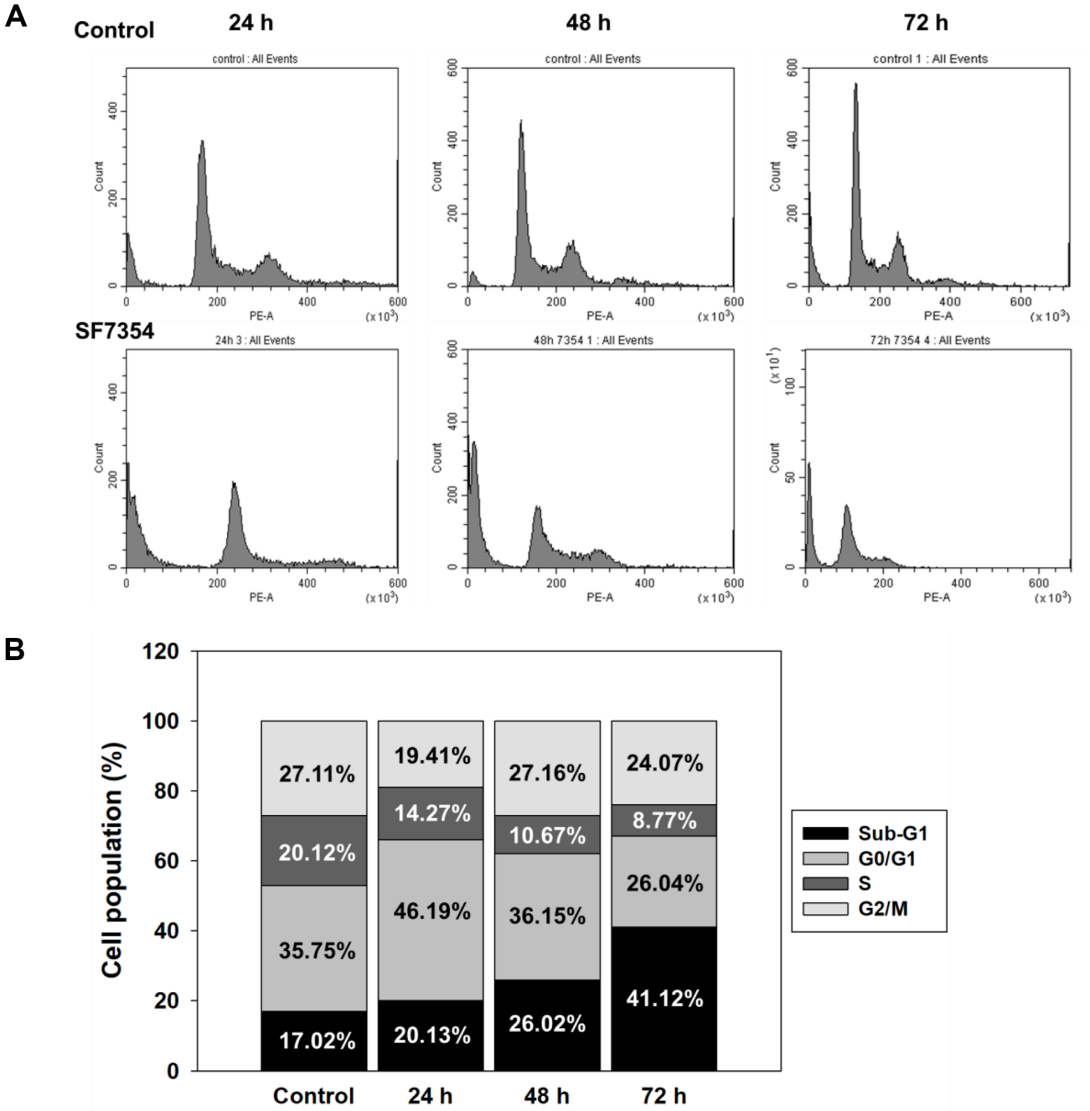
Effect of *P. spathulatum* SF7354 extract on cell cycle progression in HCT116 cells. (**A**) Flow cytometry histograms showing cell cycle distribution after treatment with 20 μg/ml of extract. (**B**) Quantification of cells in G0/G1, S, and G2/M phases. A time-dependent accumulation in Sub-G1 phase indicates DNA fragmentation.

Treatment of HCT116 cells with the SF7354 extract (20 μg/ml) resulted in distinct alterations in cell cycle distribution over time. As shown in Fig. 4, the proportion of cells in the Sub-G1 phase, indicative of apoptotic DNA fragmentation, progressively increased from 17.02% in the untreated control to 20.13%, 26.02%, and 41.12%at 24, 48, and 72 h, respectively. This accumulation in the Sub-G1 phase was accompanied by a time-dependent decrease in the S phase population, which declined from 20.12% (control) to 8.77% at 72 h.

### Physiological Changes of HCT116 Cells

**Fig. 5:**
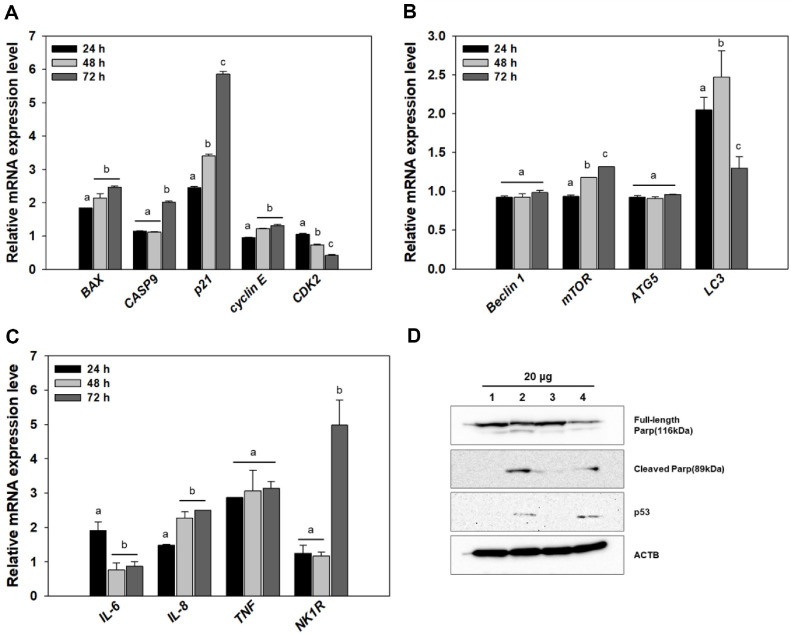
Gene and protein expression analysis of HCT116 cells treated with *P. spathulatum* SF7354 extract. Cells were treated with 20 μg/ml of extract for 24, 48, and 72 h. (**A**) Expression of apoptosis-related genes, (**B**) autophagy-related genes, and (**C**) inflammation-related genes was measured by quantitative real-time PCR (qRT-PCR). Gene expression levels were normalized to β-actin and expressed relative to the control group. Data are presented as mean ± standard error (*n* = 3). Different letters indicate statistically significant differences between time points (*p* < 0.05). (**D**) Western blot analysis of fulllength and cleaved PARP expression. Lane 1: 48 h control; Lane 2: 48 h treated; Lane 3: 72 h control; Lane 4: 72 h treated. β-actin was used as a loading control.

To elucidate the molecular response of HCT116 cells to SF7354-derived extract, the relative expression levels of genes associated with apoptosis, autophagy, and inflammatory signaling were analyzed by qRT-PCR at 24, 48, and 72 h post-treatment. Among the apoptosis-related genes, *BAX*, *CASP9*, and *p21* were notably upregulated in a time-dependent manner, with *p21* showing the most dramatic increase, especially at 72 h (5.86-fold higher than control). In contrast, the expression level of *CDK2*, a key regulator of cell cycle progression, progressively declined. Regarding autophagy, expression of *LC3* gene increased at 48 h but decreased slightly at 72 h, while *Beclin 1*, *mTOR*, and *ATG5* remained relatively stable, indicating only partial activation of the autophagy pathway. Furthermore, inflammation- and immune-related genes including *IL-6*, *IL-8*, and *TNF* were moderately elevated, whereas *NK1R* showed strong upregulation over time (Fig. 5). These results imply that the fungal extract not only induces apoptosis and modulates cell cycle dynamics but also triggers inflammatory and immunomodulatory responses in HCT116 cells.

Western blot analysis was conducted to evaluate the expression levels of apoptosis-related proteins following treatment of HCT116 cells with the extract from SF7354. The expression of full-length PARP gradually decreased in a time-dependent manner, while cleaved PARP levels remained elevated compared to the control. The protein expression level of p53 was also elevated compared to the control.

### Isolation and Identification of Anticancer Compounds from Strain SF7354

**Fig. 6:**
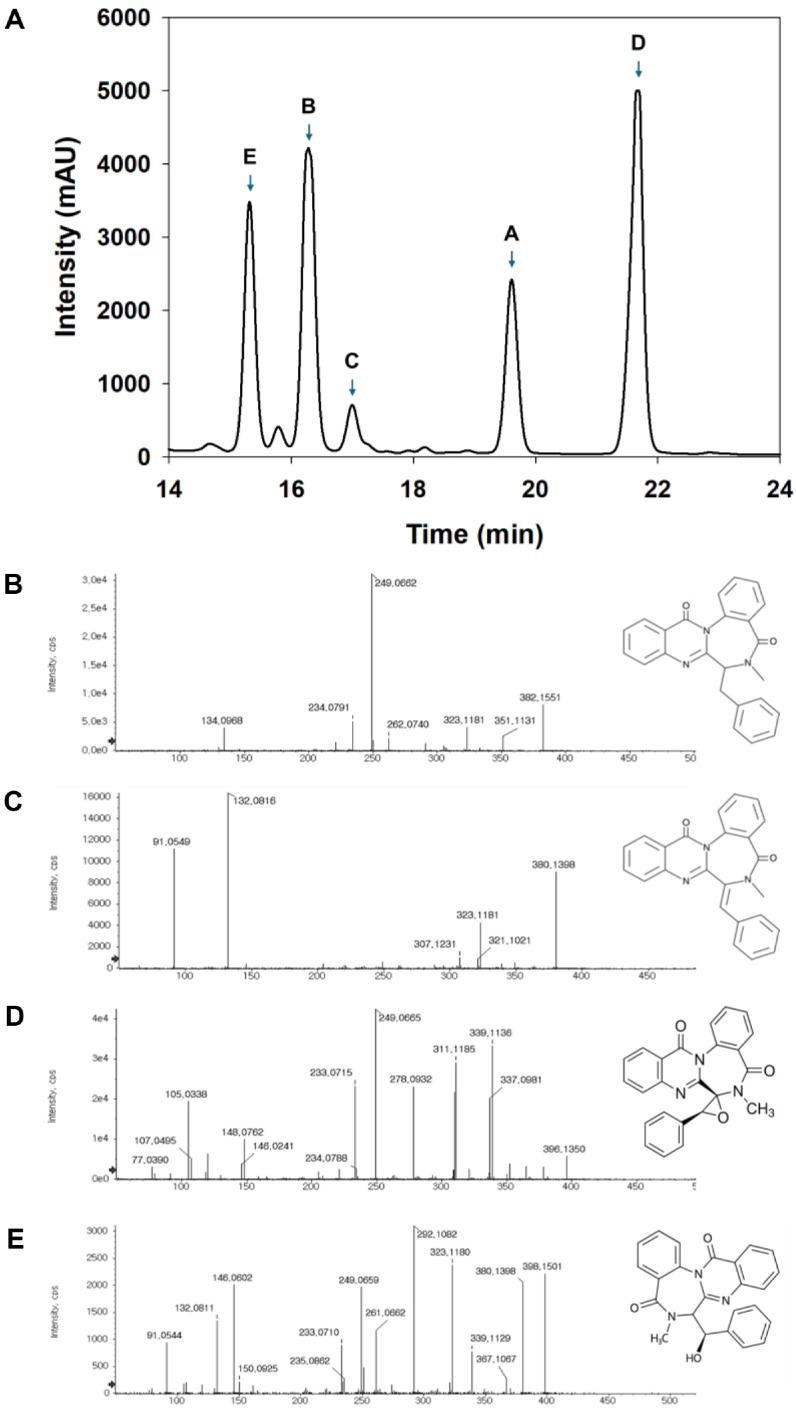
Identification of benzomalvin derivatives from *P. spathulatum* SF7354 extract. (**A**) HPLC chromatogram showing separation of five benzomalvin derivatives. MS spectra and chemical structures of the isolated compounds: (**B**) benzomalvin A/D, (**C**) benzomalvin B, (**D**) benzomalvin C, and (**E**) benzomalvin E.

**Fig. 7:**
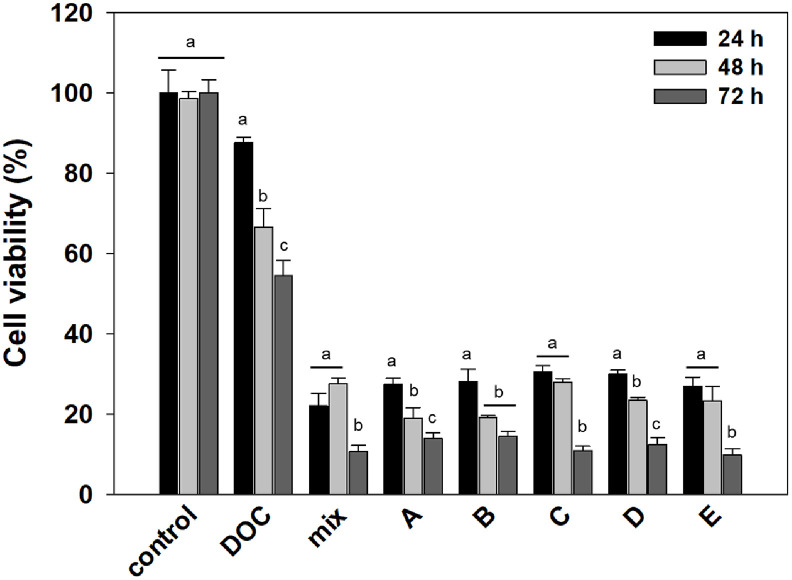
Cytotoxic activity of purified benzomalvin derivatives against HCT116 cells. Cells were treated with the mixture of fractionated extract and isolated compounds A–E (benzomalvin A, B, C, D, and E, respectively) for 24, 48, and 72 h. Cell viability was assessed by MTT assay. Control indicates the non-treated group, DOC represents the positive control (DOCtreated group), and mix refers to the crude extract-treated group. Data are presented as mean ± standard error (*n* = 3). Different letters (a-c) indicate statistically significant differences among groups (*p* < 0.01).

To identify the active anticancer components from *P. spathulatum* SF7354, the fractionated extract was further purified using a semi-preparative HPLC system (Fig. 6), resulting in the successful isolation of five single compounds: benzomalvin E (t_R = 15.32 min, 398.15 m/z), benzomalvin B (t_R = 16.28 min, 380.15 m/z), benzomalvin C (t_R = 17.00 min, 396.15 m/z), benzomalvin A (t_R = 19.61 min, 382.17 m/z), and benzomalvin D (t_R = 21.68 min, 382.17 m/z). Among the benzomalvin derivatives detected in the extract, compound C was the most abundant, accounting for 50% of the total content. Compounds B, A, and D were present at 17.6%, 14.7%, and 11.8%, respectively, while compound E showed the lowest proportion at 5.9%. As shown in Fig. 7, all five purified compounds significantly reduced the cell viability of HCT116 cells in a dose- and time-dependent manner. The IC_50_ values of each compound were 0.29, 1.88, 0.64, 1.16, and 1.07 μg/ml, respectively.

## Discussion

The results of the cytotoxicity assay demonstrate that the crude extract derived from *P. spathulatum* SF7354 exhibits broad-spectrum anticancer activity against multiple human cancer cell lines. Notably, the strongest cytotoxic effect was observed in HCT116 cells, with cell viability decreasing sharply in a dose-dependent manner. This suggests that bioactive compounds present in the extract may exert selective toxicity against HCT116 cells. The enhanced sensitivity of HCT116 compared to other cell lines, such as A549 and HeLa, could be attributed to differences in cell-specific uptake mechanisms, metabolic activity, or apoptotic signaling pathways activated by fungal metabolites [11, 12]. Given the pronounced response in HCT116 cells, this cell line was selected for subsequent mechanistic studies.

The cytotoxic effects observed in HCT116 cells following treatment with the fractionated extract from strain SF7354 suggest the presence of bioactive metabolites with potential anticancer properties. The dose- and time-dependent decrease in cell viability is indicative of a robust antiproliferative response, particularly at higher concentrations and extended exposure times. The significant reduction in viable cells, especially at 20 μg/ml after 72 h, suggests activation of programmed cell death mechanisms such as apoptosis or necrosis. Microscopic evidence further supports this finding; treated HCT116 cells exhibited characteristic morphological features of dying cells, including shrinkage and detachment [13]. These phenotypes are commonly associated with cytoskeletal breakdown and loss of adhesion, which are hallmarks of apoptosis or advanced necrotic states [14, 15].

The progressive increase in apoptotic cell populations following treatment with the extracts from *P. spathulatum* SF7354 strongly suggests the activation of time-dependent programmed cell death mechanisms in HCT116 cells. This cell death pattern may reflect the influence of secondary metabolites capable of modulating intrinsic apoptotic pathways or disrupting cellular homeostasis over time [16, 17]. Such behavior is consistent with the actions of several fungal-derived compounds known to interfere with mitochondrial function or induce oxidative stress, ultimately promoting caspase activation and membrane asymmetry [18, 19].

The perturbation of the cell cycle is a hallmark of many anticancer agents [20]. Treatment with the SF7354-derived extract appears to induce a regulated disruption of the cell cycle in HCT116 cells, favoring apoptotic processes over unregulated cytotoxicity. Notably, the increase in the sub-G1 population, a widely accepted marker for DNA fragmentation, strongly suggests activation of programmed cell death pathways [21]. Such cell cycle deregulation is commonly observed with compounds that interfere with DNA synthesis or damage response signaling, leading to checkpoint activation, particularly at the G1/S transition [22, 23].

The transcriptional analysis of HCT116 cells treated with *P. spathulatum* SF7354 extracts demonstrated coordinated activation of apoptotic, autophagic, and inflammatory signaling pathways, reflecting a multifactorial mechanism of action. Among apoptotic regulators, *BAX* and *CASP9* were significantly upregulated in a time-dependent manner, indicating the activation of the intrinsic (mitochondria-mediated) apoptotic pathway. The concurrent increase in *p21*, a cyclin-dependent kinase inhibitor known to mediate G1 arrest, further supports the notion of cell cycle checkpoint activation as a prelude to apoptosis. The increase in p53 protein levels suggests that apoptosis is mediated through p53-dependent mechanisms. This is consistent with previous studies showing that p53 activation, often triggered by oxidative stress or DNA damage, induces p21 expression and enforces G_1_/S cell cycle arrest under genotoxic conditions [24, 25, 26]. The apoptotic nature of the response was further substantiated by Western blot analysis showing robust cleavage of PARP, a hallmark of caspase-dependent apoptosis. PARP (poly ADP-ribose polymerase), a nuclear DNA repair enzyme, is cleaved by activated caspases into an 89 kDa fragment during apoptosis. These findings confirm that the SF7354 extract engages the caspase cascade.

In parallel, *LC3* expression, a marker of autophagy, showed transient elevation at 48 h. This suggests that the autophagic response may be limited or selective and functions as a complementary process to apoptosis rather than as a survival mechanism. Such crosstalk is well established, with moderate autophagy known to facilitate the removal of damaged organelles and amplify proapoptotic signaling [27, 28]. The moderate increases in *IL-6*, *IL-8*, and *TNF* also point to an inflammatory milieu, which can be associated with both apoptotic signaling and secondary immune recruitment [29, 30]. These findings collectively suggest that the fungal extract initiates a complex cell response involving apoptotic death, cell cycle inhibition, and immune-related gene expression.

The isolation of benzomalvin derivatives from *P. spathulatum* SF7354 and their significant cytotoxic effects on HCT116 cells suggest that these fungal secondary metabolites may serve as promising lead compounds for anticancer drug development. Benzomalvin derivatives, a subclass of diketopiperazine-based benzodiazepine alkaloids, are produced by *Penicillium* species and have attracted attention due to their structurally unique scaffolds and diverse biological activities. These compounds have been previously reported to exhibit neuroprotective, antimicrobial, and potential anticancer properties [31]. For instance, benzomalvin E was identified as a novel IDO inhibitor, a mechanism that may not only suppress tumor cell proliferation but also modulate the tumor immune microenvironment [32]. The dose- and time-dependent reduction in HCT116 cell viability observed in this study is consistent with earlier reports describing the antiproliferative effects of fungal-derived alkaloids [33, 34].

*P. spathulatum* SF7354, isolated from *A. monantha*, was found to produce benzomalvin-type secondary metabolites with potent anticancer activity against HCT116 cancer cells. The extract induced apoptosis and cell cycle arrest. Gene expression profiling further supported activation of intrinsic apoptotic pathways, modest autophagy, and immunoregulatory responses. These findings demonstrate that extremophile-derived fungal metabolites represent a valuable source of novel bioactive compounds and support the further investigation of benzomalvin derivatives as promising candidates for anticancer drug development.

## Supplemental Materials

Supplementary data for this paper are available on-line only at http://jmb.or.kr.
