# Supplementary material for: Anticancer Activity of Benzomalvin Derivatives Isolated from Penicillium spathulatum SF7354, a Symbiotic Fungus from Azorella monantha
Source: J Microbiol Biotechnol. 2025 Sep 16;35:e2505003. doi: 10.4014/jmb.2505.05003 (PMC12463563; doi:10.4014/jmb.2505.05003)
Supplement: Supplementary file 1 [file jmb-35-e2505003-supple.pdf]

**J. Microbiol. Biotechnol. 2025. 35: e2505003**

**https://doi.org/10.4014/jmb.2505.05003**

# Supplementary Figures and Table

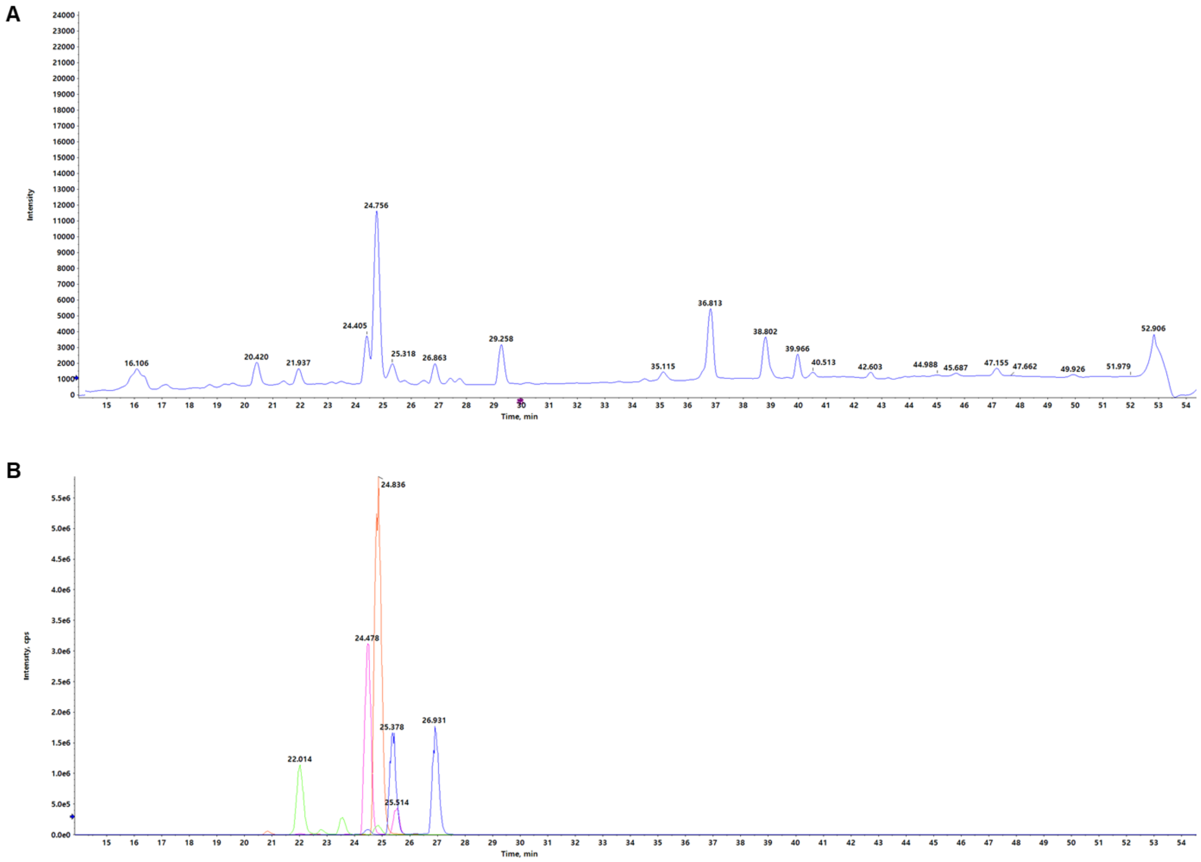


**Fig. S1. HPLC chromatogram of the crude extract from *Penicillium spathulatum* SF7354**. (**A)** UV spectrum of crude extract from SF7354. (**B)** (B) XIC analysis of the crude extract was performed to predict the retention times of benzomalvin derivatives, which were detected within the 20–30 min range.

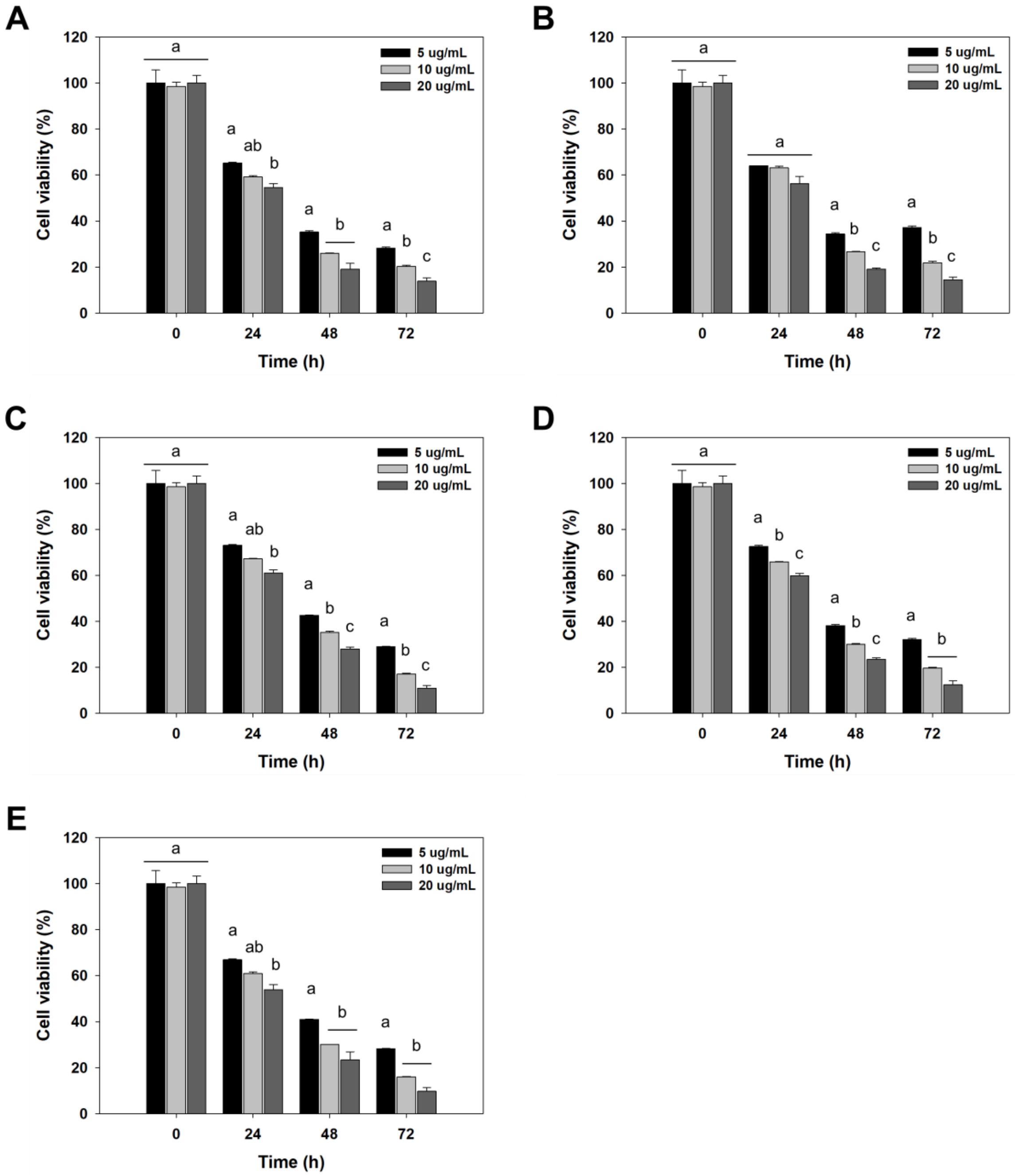


**Fig. S2. Cytotoxic activity of purified benzomalvin derivatives against HCT116 cells.**

Cells were treated with the isolated compounds A–E (benzomalvin A, B, C, D, and E, respectively) for 24, 48, and 72 h. Cell viability was assessed by MTT assay. Data are presented as mean ± standard error (n = 3).

**Table S1. Primer sequence used in this study.**

| Oligo name | Sequence |
|---|---|
| b-actin-F | TCCTCCTGAGCGCAAGTACTC |
| b-actin-R | CGGACTCGTCATACTCCTGCTT |
| BAX-F | TGGAGCTGCAGAGGATGATTG |
| BAX-R | GAAGTTGCCGTCAGAAAACATG |
| CASP9-F | CTTTGTGTCCTACTCTACTTTCC |
| CASP9-R | AACAGCATTAGCGACCCTA |
| p21-F | CATGTGGACCTGTCACTGTCTTGTA |
| p21-R | GAAGATCAGCCGGCGTTTG |
| cyclin E-F | ATCAGCACTTTCTTGAGCAACA |
| cyclin E-R | TTGTGCCAAGTAAAAGGTCTCC |
| CDK2-F | GCTAGCAGACTTTGGACTAGCCAG |
| CDK2-R | AGCTCGGTACCACAGGGTCA |
| Bec-F | GGTTGCGGTTTTTCTGGGAC |
| Bec-R | TTGATGGAATAGGAGCCGCC |
| mTOR-F | CGCGAACCTCAGGGCAA |
| mTOR-R | CTGGTTTCCTCATTCCGGCT |
| ATG5-F | GCAACTCTGGATGGGATTGC |
| ATG5-R | CAACTGTCCATCTGCAGCCA |
| LC3-F | GCGAGTTACCTCCCGCAG |
| LC3-R | GTACCTCCTTACAGCGGTCG |
| IL6-F | AACCTGAACCTTCCAAAGATGG |
| IL6-R | TCTGGCTTGTTCCTCACTACT |
| IL8-F | CATACTCCAAACCTTTCCACCCC |

| | |
|---|---|
| IL8-R | TCAGCCCTCTTCAAAAACTTCTCCA |
| TNF-F | GGAGAAGGGTGACCGACTCA |
| TNF-R | CTGCCCAGACTCGGCAA |
| NK1R-F | GCTGCCCTTCCACATCTTCT |
| NK1R-R | CCCAGACGGAACCTGTCATT |
